# Diuretics decrease fluid balance in patients on invasive mechanical ventilation: the randomized-controlled single blind, IRIHS study

**DOI:** 10.1186/s13054-021-03509-5

**Published:** 2021-03-10

**Authors:** Raphaël Cinotti, Jean-Baptiste Lascarrou, Marie-Ange Azais, Gwenhaël Colin, Jean-Pierre Quenot, Pierre-Joachim Mahé, Antoine Roquilly, Aurélie Gaultier, Karim Asehnoune, Jean Reignier

**Affiliations:** 1 CHU Nantes, Pôle Anesthésie-Réanimation, Service d’Anesthésie Réanimation Chirurgicale, Hôpital Guillaume et René Laennec, Université de Nantes, 44800 Saint-Herblain, France; 2 Médecine Intensive et Réanimation, Hôtel Dieu, University Hospital of Nantes, 1 place Alexis Ricordeau, 44093 Nantes, France; 3 Médecine Intensive et Réanimation, Centre Hospitalier Départemental de la Vendée, La Roche-sur-Yon, France; 4 Service de Médecine Intensive-Réanimation, CHU Dijon-Bourgogne, Dijon, France; 5 Equipe Lipness, Centre de Recherche INSERM UMR1231 et LabEx LipSTIC, Université de Bourgogne-Franche Comté, Dijon, France; 6 INSERM, CIC 1432, Module Épidémiologie Clinique, Université de Bourgogne-Franche Comté, Dijon, France; 7 CHU Nantes, Pôle Anesthésie-Réanimation, Service d’Anesthésie Réanimation Chirurgicale, Hôtel Dieu, Université de Nantes, 44093 Nantes, France; 8 Laboratoire UPRES EA 3826 «Thérapeutiques cliniques et expérimentales des infections», University Hospital of Nantes, 1 rue Gaston Veil, 44035 Nantes Cedex 1, France; 9 Direction de la recherche, Plateforme de Méthodologie et Biostatistique, CHU de Nantes, Nantes, France

**Keywords:** Fluid balance, Diuretic, Mechanical ventilation, Weaning

## Abstract

**Background:**

Fluid overload has been associated with increased morbidity and mortality in critically ill patients. The goal of this study was to assess the efficacy and safety of a diuretic strategy to overcome positive fluid balance in patients on invasive mechanical ventilation.

**Methods:**

Design: Multicenter, single-blind, randomized-controlled study. Patients were randomized into a diuretic (furosemide) or a control group. Patients were eligible in case of fluid overload defined as in-ICU weight increase ≥ 3%, invasive mechanical ventilation (FiO_2_ ≤ 60% and PEEP ≤ 10 cm H_2_O on inclusion) and hemodynamic stabilization. The primary outcome was fluid balance, defined as weight variation from reference weight to successful extubation. The main secondary outcome was the safety of diuretic.

**Results:**

171 patients were randomized. After 5 exclusions, 166 patients were included in the analysis: 77 in the diuretic and 89 in the control group*.* Fluid balance was 1.4 [− 2.5 to 4.5] kg in the diuretic and 6.4 [0.5–11.2] kg in the control group (*p* < 0.001). In the multiple imputation analysis, fluid balance was significantly decreased in the diuretic group (mean difference = − 4.8 95% CI [− 7.3 to − 2.5], *p* < 0.001). Eleven (14%) patients died in the diuretic group and 16 (18%) patients in the control group (*p* = 0.5). There was a worsening of Acute Kidney Injury in 67 (75.3%) patients of the control group versus 46 (59.7%) patients in the diuretic group (*p* = 0.03).

**Conclusions:**

In this multicenter randomized-controlled study, protocolized diuretic therapy reduced fluid accumulation in patients receiving mechanical ventilation and was well tolerated with a favorable safety profile.

*Trial registration* NCT 02345681, Registered January 26 2015, Prospectively registered, https://clinicaltrials.gov/ct2/show/NCT02345681?term=02345681&draw=2&rank=1.

**Supplementary Information:**

The online version contains supplementary material available at 10.1186/s13054-021-03509-5.

## Background

Positive fluid balance has been associated with worse outcome in critical care patients (sepsis [1, 2], burns [3], major surgery [4], acute kidney failure [5] and in critically ill children [6]). Fluid balance is induced by systemic inflammatory response syndrome, volume expansion and cardiac and kidney failure and is present in the vast majority of patients [1, 7].

Both fluid restriction and diuretics have been proposed for the prevention and treatment of patients with fluid overload in the early time phase. In patients with acute respiratory distress syndrome, an early multimodal strategy combining restrictive fluid management and diuretics at the initial phase, was associated with a reduction of the duration of mechanical ventilation [8]. A restrictive fluid management strategy during the initial care of sepsis was associated with reduced volumes of fluids, compared with standard care [9]. During the post-acute phase, natriuretic peptide-driven fluid management during ventilator weaning was associated with decreased duration of weaning [10]. However, in the stabilization phase of critical care illness, the efficacy and safety of systematic diuretics to decrease positive fluid balance has not been thoroughly investigated. Moreover, no guidelines are available regarding the timing and indication of diuretics from weaning of mechanical ventilation in ICUs.

We hypothesized that the administration of diuretics can decrease the fluid balance in patients with weight gain undergoing invasive mechanical ventilation. We therefore tested this hypothesis in the single-blind randomized-controlled multicenter IRIHS study.

## Methods

### Participants

This was a pragmatic multicenter, single-blind, randomized-controlled study, in 4 ICUs in 3 French hospitals (Dijon, Nantes, La Roche sur Yon), from May 5 2015 to February 2 2019 (NCT 02345681). The IRIHS study was performed in accordance with CONSORT guidelines (Additional file 1). The study was approved by an ethics committee (Comité pour la Protection des Personnes Ouest IV Nantes—IRB N° 41/14). Whenever possible, patients received oral and written information and provided written consent. When the patient was unable to consent, next-of-kin received information and provided written consent prior to inclusion. Retrospective consent was obtained from patients, as soon as deemed possible. The research staff monitored the study for integrity and quality of data.

### Inclusion criteria

Patients ≥ 18 years old, admitted to an ICU undergoing mechanical ventilation (FiO_2_ ≤ 60% and PEEP ≤ 10 cm H_2_O on inclusion), and displaying positive fluid balance were included after hemodynamic stabilization [11]. Hemodynamic stabilization was defined as follows: discontinuation of vasopressor drugs for at least 6 h and/or dobutamine infusion ≤ 10 μg kg^−1^ min^−1^. Patients were assessed for eligibility when undergoing invasive mechanical ventilation and vasoactive drugs were stopped. Patient weight was assessed daily in order to obtain baseline weight, weight on inclusion, daily weight after randomization and at extubation. After successful extubation, weight was no longer monitored for the protocol. When fluid balance was ≥ 3%, informed consent was obtained from next-of-kin. In a study regarding vascular expansion with vascular expansion in septic shock patients [11], the lowest ratio between vascular expansion and patient body weight to detect a positive fluid balance was 3% and was therefore maintained in our study. Initially, the reference weight was set at 24 h after ICU admission, in order to avoid potential interaction between weight and hypovolemia or blood loss. However, owing to early positive fluid balance after initial resuscitation, criteria were modified on April 6, 2016 (IRB approval MS3), and the reference weight was set on ICU admission instead of 24 h after hospitalization. Initially, patients were included in case of invasive mechanical ventilation on admission or in the first 24 h after admission. After substantial modification (April 6, 2016, IRB approval MS3), patients could be included if they underwent invasive mechanical ventilation during ICU stay.

### Exclusion criteria

Patients were not eligible in case of pregnancy, withdrawal of life-sustaining therapies in the 24 h after admission, allergy to furosemide, admission for decompensated cirrhosis, hospitalization for central neurologic injury (trauma, intra-cranial hemorrhage, spinal injury), chronic kidney failure (creatinine clearance ≤ 30 mL min^−1^ or dialysis), and when the diuretic treatment was mandatory (acute pulmonary edema, heart failure with a reduced ejection fraction ≤ 30%).

### General ICU management

Vascular expansion was performed according to standardized protocol guided by echocardiography [12, 13], pulse pressure variation [14] and clinical signs of acute circulatory failure [15]. Management of analgesia and sedation was performed using validated clinical scales (Richmond Assessment Sedation Scale [16], Behavior Pain Scale [17]).

Weaning from mechanical ventilation was performed according a predefined protocol. After discontinuation of sedation, patients were checked for extubation at least once a day according to predefined weaning criteria [18]: stable cardiovascular status (heart rate 140 beats/min or less, systolic blood pressure 90 to 160 mmHg, and minimal or absence of catecholamine), adequate oxygenation (oxygen saturation measured by pulse oximetry ≥ 90%, fractional inspired oxygen tension ≤ 40%, positive end-expiratory pressure ≤ 8 cm H_2_O, respiratory rate ≤ 35 breaths/min), PaCO_2_ ≤ 50 mmHg, core temperature ≤ 38.5 °C, no agitation. Spontaneous breathing trials were systematically performed in patients when all weaning criteria were achieved. Patients who completed successful spontaneous breathing trials were extubated. Successful extubation was defined as endotracheal tube removal for at least 48 h [18].

### Randomization

The randomization sequence was generated by a statistician at the clinical research unit (CHU Nantes) who had no role in patient recruitment. The randomization scheme was performed in blocks of 6, balanced (1:1 ratio) and stratified by center, chronic treatment by diuretic or not, and episode of acute kidney injury or not, defined as extra-renal replacement therapy or a creatinine blood level ≥ 180 μmol L^−1^ [10]. The software used to collect the data from the electronic report form automatically allocated the patients. Randomization was performed through a web-based system according to the IWRS system. The IRIHS study was single-blinded. Patients and relatives were not aware of the arm of randomization.

### Intervention

In the diuretic group, patients were given furosemide once or twice a day until successful extubation. The dosage was adapted by the bedside physician with the aim to reach patient’s reference weight, with a maximum daily dose set at 250 mg of furosemide to avoid kidney toxicity [19]. The administration of diuretics was postponed if one of the following criteria was present: urea > 25 mmol L^−1^, creatinine > 180 μmol L^−1^, creatinine clearance ≤ 30 mL min^−1^, administration of iodinated contrast product in the past 6 h, natremia > 150 mmol L^−1^, kalemia < 3 mmol L^−1^, pH > 7.50, bicarbonates > 40 mmol L^−1^ [10]. Once these metabolic issues were corrected, diuretics could be administered. After successful extubation, diuretic management was left to the attending physician’s discretion.

### Control

In the control group, diuretic administration was prohibited. Diuretic could be used as rescue therapy in case of acute pulmonary edema or de novo heart failure.

### Outcomes

#### Primary outcome

The primary outcome was the patient’s fluid balance. Fluid balance was defined as the difference between the body weight assessed at the time of extubation and the body weight assessed on randomization.

#### Secondary outcome: efficacy criteria

The rate of extubation failure [18], the duration of invasive mechanical ventilation from randomization to successful weaning, the number of ventilatory free-days by day-28, the duration of ICU stay, ICU mortality and mortality by day-60 were compared between groups.

#### Secondary outcome: safety criteria

We compared the incidence and duration of hypokalemia episodes (≤ 3.5 mmol L^−1^), the incidence and duration of hyponatremia (≤ 135 mmol L^−1^), the number of hypernatremia episodes (≥ 145 mmol L^−1^), the rate of cardiac arrythmia (atrial fibrillation, torsade de pointes, ventricular tachycardia), the rate of kidney injury (KDIGO classification [20]) from randomization to successful extubation. Urine output was measured daily and incorporated in the KDIGO definition. Safety events are described.

### Sample size calculation

In a multicenter randomized-controlled study of sepsis [7], the median fluid balance during resuscitation was + 5 kg. We hypothesized that in the diuretic group, the fluid balance would be nil, whereas the fluid balance would be + 5 kg in the control group. Considering a standard deviation of 10, we calculated that we would need 172 patients (86 per group) for the study with an 80% power and a one-sided level of 0.05.

### Funding source

This protocol was funded by a grant from the French Ministry of Health (Call for tenders: Roche sur Yon, Nantes, 31 January 2014). The FMH had no role in the design or conduct of the study, data collection, analysis or interpretation, the writing of the report or in the decision to submit for publication. The corresponding authors had full access to all of the data and the final responsibility to submit for publication.

### Statistical analysis

As planned, analyses were performed on all randomized patients meeting the study inclusion and non-inclusion criteria (Modified Intention to Treat Population). We also provide an Intention-to-treat analysis and a per-protocol analysis (defined as patients with an exposure to the intervention (furosemide), with available primary variable measurements and the absence of protocol violations) for the primary outcome, as a post hoc analysis.

For the treatment of missing data, a multiple imputation (Generates Multivariate Imputations by Chained Equations) based on patient characteristics at baseline (SAPS II, acute renal failure, chronic diuretic therapy, chronic renal failure, heart failure, admission to ICU for septic shock and admission to ICU for acute respiratory failure) were performed in the primary endpoint analysis.

Two simple imputation methods (worst-case and last observation carried forward imputation) and a complete analysis of cases were also explored for the primary endpoint. The worst-case scenario consisted in imputing the missing data from the control group for the smallest value of the primary endpoint (difference between the body weight assessed at the time of extubation and the reference body weight) of the patients in the control group, and the missing data of the intervention group, by the greatest value of the primary endpoint of patients in the intervention group. In patients still intubated on day-28 we compared the weight variation from randomization to day 7.

No data imputation was performed for the analysis of the secondary endpoints.

All of the variables were described globally and in the two groups, by the frequencies and percentages of each modality for the qualitative variables, and by their mean and standard deviation, or median and first and third quartile for the quantitative variables.

For analysis of the primary and the secondary endpoints, generalized linear mixed models were performed to adjust analyses on the stratification factors of randomization.

We explored the correlation between weight from extubation to randomization and output (diuresis) and intakes (colloids + cristalloids), with a Pearson test. Ventilatory-free days were calculated as the number of days between D1 and D60 when the patient was alive and not intubated. Patients were considered intubated, when they were extubated but re-intubated within 48 h. For patients who died between D1 and D60, VFD = 0 [21].

All analyses were performed with R 3.6.0 software®.

## Results

### Study population

**Fig. 1:**
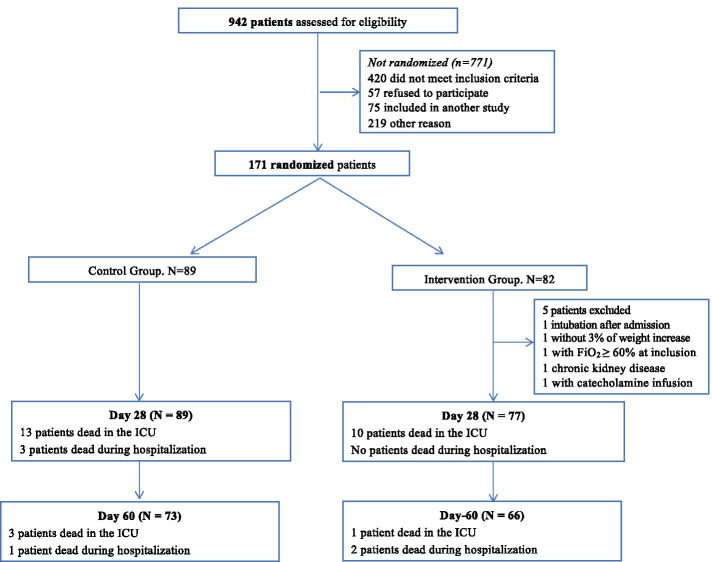
Study flowchart

There were 979 patients screened during the study period of whom 171 were randomized. Inclusions were stopped before reaching the expected 172 patients, owing to a slow inclusion rate and end of funding. Eighteen patients were included in each arm before the modification of inclusion criteria. Finally, 89 patients were included in the control group and 82 in the intervention group. After excluding five patients from the intervention group owing to inadequate inclusion criteria, 77 patients were included in the intervention group, the modified intention-to-treat population. Figure 1 describes the inclusions and follow-up. Patients were admitted for acute respiratory failure [75 (45.2%), sepsis or septic shock (49 (29.5%)], trauma [13 (7.8%), hemorrhagic shock (9 (5.4%)]. Patients were randomized 6 (4–9) days after admission. After randomization, patients received a total cumulative dose of 100 [40–160] mg of furosemide in the control group as rescue therapy according to the protocol and 160 [80–285] mg in the diuretic group (*p* < 0.001). Patient characteristics are described in Table 1.

**Table 1:** Baseline characteristics

	Control group *N* = 89	Diuretic group *N* = 77
Age	66 [60–74]	66 [58–72]
Sex ratio F/M	28(31.5%)/61(68.6%)	16(20.8%)/61(79.2%)
SAPS II	53 [45–59]	52 [41–58]
SOFA baseline	7 [5–10]	8 [6–10]
SOFA at randomization	4 [2–6]	4 [3–5]
Height (cm)	169 [163–175]	171 [163–175]
Weight at baseline (kg)	76 [64–85]	82 [68–92]
Weight at randomization (kg)	84 [75–97]	88.5 [73–99]
MV before randomization (days)	5 [4–8]	6 [4–8]
BMI	25.5 [22.6–29.2]	28.4 [25.5–34.5]
Hypertension	13 (14.6%)	5 (6.5%)
Chronic respiratory disease	10 (11.2%)	5 (6.5%)
Chronic kidney disease	3 (3.4%)	1 (1.3%)
Use of diuretics	11 (12.4%)	8 (10.4%)
Diabetes mellitus	9 (10.1%)	11 (14.3%)
Active smoking	21 (23.6%)	14 (18.2%)
Admission		
Sepsis/septic shock	28 (31.4%)	21 (27.2%)
Acute respiratory failure	42 (47.2%)	33 (42.9%)
Trauma	7 (7.9%)	6 (7.8%)
Hemorrhagic shock	5 (5.6%)	4 (5.2%)
Elective surgery	1 (1.1%)	4 (5.2%)
Misc	6 (6.8%)	9 (11.7%)
RRT/AKI before randomization	27 (30.3%)	21 (27.3%)

*N* (%) for qualitative variable and median [Q1–Q3] for quantitative variable

*BMI* body mass index, *RRT* renal-replacement therapy, *MV* mechanical ventilation, *AKI* acute kidney injury defined as a blood level of creatinine ≥ 180 μmol L^−1^

### Primary outcome: fluid balance

**Fig. 2:**
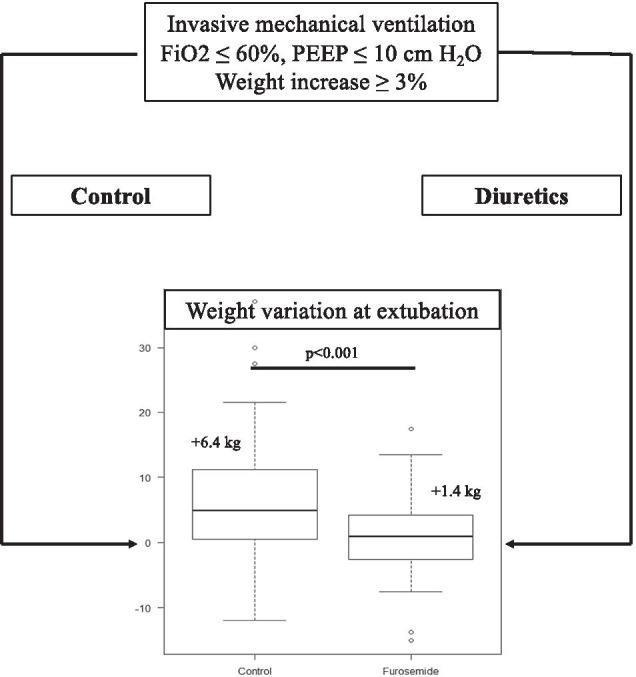
Design and main result

Fluid balance was not available in 22 patients (7 patients died without extubation, 12 patients without extubation before the end of follow-up on day-28 and 3 patients owing to investigator failure). In the multiple imputation analysis, fluid balance was lower in the intervention group (mean difference = − 4.8 CI_95_ [− 7.3 to − 2.5], *p* < 0.001). In the complete case analysis (144 (86.7%) patients), the median fluid balance was 1.4 [− 2.5 to 4.5] kg in the diuretic group, and 6.4 [0.5–11.2] kg in the control group (*p* < 0.001) (Fig. 2). With the worst-case scenario imputation [− 12 in the control group (14 patients) and + 17 in the intervention group (8 patients)], this difference was no longer significant (1.5 [− 2 to 7.7] vs 4.5 [− 1.5 to 10.5] kg, *p* = 0.7). In the per protocol analysis (*N* = 135), fluid balance was significantly lower in the intervention group (1 [− 2 to 4.6] kg vs 5 [0.4–11] kg, *p* < 0.001). Data regarding fluid balance are available in Table 2. In the post hoc intention-to-treat analysis, fluid balance was significantly lower in the intervention group for the multiple imputation analyses (β = − 4.7 CI_95_ [− 7.0 to − 2.4], *p* < 0.001) and the complete case (*N* = 147) (1.0 [− 2.5 to 4.1] vs 5.0 [0.5–11.2], *p* < 0.001). Finally, in the last observation carried forward exploratory analysis, weight variation was significantly lower in the intervention group (*p* = 0.047, Table 2). In patients still intubated on day-28, weight variation was not significantly different between the 2 groups (11.3 [8.8–16.9] kg in the control group vs 6 [0.9–10.2] in the intervention group, *p* = 0.2).

**Table 2:** Evolution of fluid balance between groups

	Control group *N* = 89	Intervention group *N* = 77	Mean difference 95% CI	*p* value
Primary analysis				
Multiple imputation (*N* = 166)			− 4.9 [− 7.4;− 2.5]	< 0.001
Sensitivity analyzes				
Complete cases (*N* = 144, 86.7%)	6.4 [5–11.2] kg	1.4 [1–4.5] kg	− 5.1 CI_95_ [− 7.4;− 2.8]	< 0.001
Worst case imputation (*N* = 166)	4.5 [− 1.5;10.5] kg	1.5 [− 2;7.7] kg	− 0.5 [− 3.2;2.3]	0.7
Last observation carried forward (*N* = 166)	7 [2;15.5] kg	1.5 [− 2;7.7] kg	− 8.4 [− 16.6;− 0.2]	0.047
Per protocol (*N* = 135, 81.3%)	5 [0.5;11.3] kg	1 [− 2.0;4.6] kg	− 4.7 [− 7.1;− 2.3]	< 0.001

Evolution of fluid balance between successful extubation and randomization. A multiple imputation was performed (SAPS II, acute renal failure, chronic diuretic therapy, chronic renal failure, heart failure, admission to ICU for septic shock and admission to ICU for acute respiratory failure). This table displays the median [quartile1–quartile3] value of measured body weight in kilograms, between the two groups (this data is not available for multiple imputation, which uses multiple imputed datasets) and generalized linear mixed models results (estimated mean difference and its confidence interval)

### Secondary outcomes

The total duration of invasive mechanical ventilation was 12 [8–21] days in the intervention group and 14 [8–22] days in the control group. The duration of mechanical ventilation from randomization to extubation was 6 [2–14] days in the intervention group and 7 [3–17] days in the control group. The number of ventilatory-free days on day-60 was 54 [37–57] days in the intervention group and 51 [32–56] days in the control group. There was a significant correlation between weight and diuresis (*r*^2^ = − 0.43, *p* < 0.001), but not intake and weight. In the diuretic group, 11 (14%) patients died in ICU as compared with 16 (18%) patients in the control group (Table 3).

**Table 3:** Outcomes

	Control group *N* = 89	Intervention group *N* = 77	Mean difference 95% CI	*p* value
*Mechanical ventilation (d)*				
Overall duration	14 [8–22]	12 [8–21]	− 0.6 [− 3.6 to 2.4]	0.7
After randomization	7 [3–17]	6 [2–14]	− 2.4 [− 6 to 1.2]	0.2
VFD at day-60	51 [32–56]	54 [37–57]	4.0 [− 2.6 to 10.6]	0.3
Extubation failure	11 (15.3%)	6 (9%)	0.5 [0.2–1.5]	0.3
RRT after randomization	4 (4.5%)	6 (7.8%)	1.8 [0.5–7.3]	0.4
In-ICU LOS (d)	18 [10–32]	18 [11–29]	− 1.9 [− 6.3 to 2.5]	0.4
In-ICU mortality	16 (18%)	11 (14%)	0.8 [0.3–1.9]	0.5
Hospital LOS (d)	36 [22–55]	32 [18–53]	− 2.0 [− 11.5 to 7.3]	0.6
Mortality at day-60	20 (22.5%)	13 (16.9%)	1.5 [0.7–3.4]	0.5

*VFD* ventilatory free days, *ICU* intensive care unit, *LOS* length of stray, *RRT* renal replacement therapy

### Safety criteria

Regarding metabolic complications, the rate of episodes of hypokalemia was 68.8% in the intervention group and 57.3% in the control group. The duration of these episodes was 1 [0–4] day in the intervention group and 1 [0–2] day in the control group. The rate of atrial fibrillation was 17.1% in the diuretic group and 19.1% in the control group. After randomization, there was a worsening of acute kidney injury in the control group (67 (75.3%) compared to the intervention group 46 (59.7%), *p* = 0.0.3) (Table 4). Safety data are described in Table 4. Safety events are provided in the Additional file 2.

**Table 4:** Safety outcomes

	Control group *N* = 89	Intervention group *N* = 82	*p* value
*KDIGO (N)*			
KDIGO 0	1 (1.1%)	0	0.9
KDIGO 1	51 (58.6%)	53 (69.7%)	0.2
KDIGO 2	6 (6.9%)	4 (5.3%)	0.7
KDIGO 3	29 (33.3%)	19 (25.0%)	0.3
Worsening of AKI	67 (75.3%)	46 (59.7%)	0.03
Natremia ≤ 135 mmol/L (N)	42 (47.2%)	33 (42.9%)	0.7
Natremia ≥ 145 mmol/L (N)	40 (44.9%)	40 (52%)	0.5
Episode of hypokalemia (N)	51 (57.3%)	53 (68.8%)	0.1
Duration of hypokalemia (d)	1 [0–2]	1 [0–4]	0.2
*Cardiac rhythm troubles (N)*			
Atrial fibrillation	14 (15.3%)	9 (11.7%)	0.5
Torsade de pointes	0	1 (1.3%)	0.5
Ventricular tachycardia	2 (2.3%)	2 (2.6%)	0.9
Ventricular fibrillation	2 (2.3%)	1 (2.6%)	0.9

Hypokalemia (≤ 3.5 mmol L^−1^)

*RRT* renal replacement therapy

## Discussion

In the multicenter randomized-controlled single blind IRIHS study, diuretics decreased fluid balance. No cardiac or renal safety issues were recorded. Nevertheless, we did not demonstrate an improvement in the outcome of our patients.

In various critical care settings ([1–6]), fluid balance has been systematically linked with poor outcome and prolonged mechanical ventilation [22], which advocates for better control of fluid balance in the ICU. However, there is little data in the current literature regarding potentially effective strategies. In a multicentric cohort of patients undergoing renal replacement therapy, a negative fluid balance was associated with better outcomes [23]. In patients with acute respiratory distress syndrome, an early restrictive fluid strategy encouraging the use of diuretics, was associated with a significant increase in the duration of ventilatory-free days [8]. Eventually, furosemide significantly decreased the duration of weaning from mechanical ventilation, but with modest clinical impact [10]. Positive fluid balance remains highly common [24] in patients undergoing invasive mechanical ventilation and is frequently the consequence of exogenous intake (medication, maintenance fluids) [25]. Our study confirms that diuretics could easily decrease fluid balance, with good cardiac and kidney safety.

In the literature, there are concerns regarding the safety of diuretics in the ICU. In a multicentric cohort of patients with acute kidney injury [19], the use of diuretics was associated with increased mortality by day-60. However, in more recent cohorts [26, 27], diuretics were associated with decreased mortality and improved kidney function. Thus, the use of diuretics in the ICU to control fluid balance could be promising, but there is little evidence regarding the benefit/risk ration of this medication in critically ill patients. Our study included severe patients with organ dysfunctions and diuretics were administered to overcome fluid balance in intubated patients with positive fluid balance. The effects of diuretics to counterbalance positive fluid balance could therefore be promising.

We decided to perform a randomized-controlled study focused on controlling fluid balance. Our aim was to validate the concept that diuretics could efficiently overcome fluid balance without major side effects. Thus, the choice of fluid balance as a primary outcome is questionable since it is not a robust endpoint in critical care literature. However, it would appear to be mandatory as a first step to validate the concept and then perform larger studies on patient outcome. Moreover, our randomized-controlled design enabled a more robust evaluation of diuretic side-effects in critically ill patients. Achieving an overall negative fluid balance could require other strategies (fluid restriction, increased furosemide doses, starting furosemide earlier). Finally, a randomized-controlled trial promoting care targeting a negative fluid balance strategy in order to improve robust endpoints such as duration of mechanical ventilation or mortality could be performed, given the promising data of this first study. However, a double-blind design could be illusory because of the strategies tested (effects of furosemide on diuresis and fluid restriction).

## Limitations

Our study was neither double-blind nor placebo-controlled. Our open design could have overestimated the effect of our intervention. A double-blind placebo-controlled study would have limited this aspect. However, we believed that the effect of furosemide on diuresis could have rendered a double-blind study rather theoretical since urine output was much higher in the diuretic group and we chose an open design in this pilot study. Owing to stratification, there was an imbalance between the two groups regarding the number of patients. Stratification should be limited in order to avoid imbalance, which could have ultimately decreased the power of the study. During the study period, more patients were admitted in our ICUs but we did not screen patients with non-inclusion criteria such as neurologic patients. We chose to include patients with moderate respiratory failure, but patients with severe respiratory failure could benefit from diuretics. The timing between intubation and randomization was variable in our study. This aspect could have influenced of our strategy on major outcomes (ventilatory-free days, mortality) but did not appear to be problematic in the evaluation of fluid balance control. Obtaining a patient’s body weight is easy to perform every day. Owing to the major catabolism occurring after a severe organ dysfunction [28] patients quickly present sustained and unavoidable protein and weight loss [29, 30]. A patient’s body weight in the ICU is therefore a marker of fluid balance and not muscle increase. We did not record the stages of acute kidney injury before randomization, apart from the presence of a renal replacement therapy. KDIGO classification was assessed only from randomization to successful extubation, but we do not have further data out of this time frame.

## Conclusion

In this multicenter randomized-controlled study, an easy-to-use clinical parameter (weight gain) to guide diuretic administration decreased fluid balance in patients undergoing invasive mechanical ventilation. The safety of diuretics regarding cardiac and metabolic issues was good. However, the control of fluid balance with diuretics in order to improve the outcome remains to be demonstrated.

## Supplementary Information

**Additional file 1**. CONSORT check-list.

**Additional file 2**. Safety events in the IRIHS study.

## Data Availability

The datasets used and/or analyzed during the current study are available from the corresponding author on reasonable request.

## Abbreviations

ICU: Intensive care unit
OR: Odds ratio
CI: Confidence of interval
