# Supplementary material for: Diuretics decrease fluid balance in patients on invasive mechanical ventilation: the randomized-controlled single blind, IRIHS study
Source: Crit Care. 2021 Mar 10;25:98. doi: 10.1186/s13054-021-03509-5 (PMC7943707; doi:10.1186/s13054-021-03509-5)
Supplement: Supplementary file 2 — Additional file 2. Safety events in the IRIHS study. [file 13054_2021_3509_MOESM2_ESM.docx]

**Additional file 2.**

|  | **Control group** | **Furosemide Group** | **Total** |
| --- | --- | --- | --- |
| **Blood and lymphatic system disorders** | 1 | 2 | 3 (5,1%) |
| Anemia | 1 | 1 | 2 (3,4%) |
| Thrombocytopenia |  | 1 | 1 (1,7%) |
| **Cardiac disorders** | 3 | 1 | 4 (6,8%) |
| Atrioventricular block complete | 1 |  | 1 (1,7%) |
| Cardio-respiratory arrest | 2 | 1 | 3 (5,1%) |
| **Gastrointestinal disorders** | 1 | 1 | 2 (3,4%) |
| Intestinal dilatation | 1 |  | 1 (1,7%) |
| Intra-abdominal hematoma |  | 1 | 1 (1,7%) |
| **General disorders and administration site conditions** | 2 |  | 2 (3,4%) |
| Multiple organ dysfunction syndrome | 2 |  | 2 (3,4%) |
| **Infections and infestations** | 9 | 5 | 14 (23,7%) |
| Abdominal wall abscess | 1 |  | 1 (1,7%) |
| Clostridium difficile infection |  | 1 | 1 (1,7%) |
| Device related infection | 1 |  | 1 (1,7%) |
| Nosocomial infection | 1 |  | 1 (1,7%) |
| Pneumonia | 3 | 1 | 4 (6,8%) |
| Purulence | 1 |  | 1 (1,7%) |
| Scrotal abscess |  | 1 | 1 (1,7%) |
| Septic shock | 1 | 1 | 2 (3,4%) |
| Skin candida |  | 1 | 1 (1,7%) |
| Urinary tract infection | 1 |  | 1 (1,7%) |
| **Metabolism and nutrition disorders** | 5 | 9 | 14 (23,7%) |
| Hypernatremia |  | 5 | 5 (8,5%) |
| Hypokalemia | 3 | 1 | 4 (6,8%) |
| Hyponatremia |  | 1 | 1 (1,7%) |
| Metabolic alkalosis |  | 2 | 2 (3,4%) |
| Metabolic disorder | 1 |  | 1 (1,7%) |
| Starvation | 1 |  | 1 (1,7%) |
| **Nervous system disorders** | 1 | 2 | 3 (5,1%) |
| Neuromyopathy | 1 | 1 | 2 (3,4%) |
| Neuropathy peripheral |  | 1 | 1 (1,7%) |
| **Renal and urinary disorders** |  | 2 | 2 (3,4%) |
| Renal failure |  | 2 | 2 (3,4%) |
| **Reproductive system and breast disorders** |  | 1 | 1 (1,7%) |
| Prostatitis |  | 1 | 1 (1,7%) |
| **Respiratory, thoracic and mediastinal disorders** | 5 | 3 | 8 (13,6%) |
| Acute pulmonary edema |  | 1 | 1 (1,7%) |
| Acute respiratory distress syndrome | 1 |  | 1 (1,7%) |
| Laryngeal edema | 2 |  | 2 (3,4%) |
| Pleural effusion |  | 1 | 1 (1,7%) |
| Pneumothorax | 1 |  | 1 (1,7%) |
| Pulmonary embolism | 1 |  | 1 (1,7%) |
| Pulmonary fibrosis |  | 1 | 1 (1,7%) |
| **Skin and subcutaneous tissue disorders** | 1 | 1 | 2 (3,4%) |
| Dermatitis exfoliative generalised |  | 1 | 1 (1,7%) |
| Livedo reticularis | 1 |  | 1 (1,7%) |
| **Vascular disorders** | 2 | 2 | 4 (6,8%) |
| Aortic thrombosis | 1 |  | 1 (1,7%) |
| Hypotension |  | 2 | 2 (3,4%) |
| Inferior vena cava dilatation | 1 |  | 1 (1,7%) |
| **Total** | 30 | 29 | 59 (100%) |
